# 

**Published:** 1826-05

**Authors:** 


### MONTHLY CATALOGUE OF MEDICAL BOOKS.

*** *It having been hinted to us that gentlemen, sending copies of their Works, prefer having their titles given at length, it is our intention, in future, to comply with this suggestion: but it is to be observed, that no books can be entered on this List except those sent to us for the purpose;—finding, in the lists hitherto transmitted, that the names of works have frequently been given as published, which have not appeared for weeks, or even months, after.*

Practical Observations on the Convulsions of Infants. By JOHN NORTH, Surgeon Accoucheur, Member of the Royal College of Surgeons.—London, 1826.

Observations on M. LAENNEC's Method of forming a Diagnosis of the Diseases of the Chest by means of the Stethoscope, and of Percussion; and upon some Points of the French Practice of Medicine. By CHARLES SCUDAMORE, M.D. F R.S. Member of the Royal College of Physicians in London; Honorary Member of Trinity College, Dublin, &c. &c.—London, 1826.

The Anatomy of the Brain, with a general View of the Nervous System. By G. SPURZHEIM, M.D. of the Universities of Vienna and Paris; Licentiate of the Royal College of Physicians in London. Translated from the unpublished French MS. by R. WILLIS, Member of the Royal College of Surgeons in London. With eleven Plates.—London, 1826.

Doubts of Hydrophobia, as a specific Disease, to be communicated by the Bite of a Dog; with Experiments on the supposed Virus generated in that Animal, during the Complaint termed Madness. By ROBERT WHITE, Surgeon, Brighton. —London, 1826.

Observations on the prevailing Practice of supplying Medical Assistance to the Poor, commonly called the Farming of Parishes; with Suggestions for the Establishment of Parochial Medicine Chests, or Infirmaries in Agricultural Districts. —London.

Thoughts on Medical Education, and a Plan for its Improvement: addressed to the Council of the University of London.—London, 1826.

Remarks on the present State of the Medical Profession, showing chiefly the Necessity for the Division of Labour in its Practice. By LEONARD STEWART, M.D. Licentiate of the Royal College of Physicians, Fellow of the Royal Medical Society of Edinburgh, and Secretary for Foreign Correspondence to the Medical Society of London.—London, 1826.

---

*** We have received a Plate of the Eye, "engraved by J. STEWART, from a Drawing by A. G. ROWLANDS, after the Plates of ZINN and SOEMMERING, and used at Mr. H. MAYO's Lecture-room, Berwick-street."—It is a splendid engraving.

### NOTICE TO CORRESPONDENTS.

*We beg to inform* Dr. VENABLES, *that his Paper has been unavoidably postponed, from circumstances connected with the printing, and not dependent upon the Editors.*

Dr. STOKES'S *Communication came to hand too late for the present month.*

Dr. LEE'S *Account of the Scurvy in Russia, and the accompanying Paper, have been received.*

*The "Clinical Report" in our next.*

*** *We have received the Titles of some Books, with a request to announce their publication. This belongs to the Advertising department, and all such Communications must be forwarded to the Publisher. Books sent to the Editor will always be duly acknowledged in the Monthly List of Publications.*

